# Posterior Cervical Fixation: Accuracy and Safety of Free-Hand Inserted Screws

**DOI:** 10.1227/neuprac.0000000000000273

**Published:** 2026-07-23

**Authors:** Veera Kivistö, Eero Ylipulli, Heikki Mäntymäki, Antti Ronkainen, Juhana Frösen, Tuomo Thesleff

**Affiliations:** 1 Faculty of Medicine and Health Technology, Tampere University, Tampere, Finland;; 2 Department of Neurosurgery, Tampere University Hospital, Tampere University, Tampere, Finland

**Keywords:** Cervical spine fixation, Lateral mass screw, Pedicle screw

## Abstract

**BACKGROUND AND OBJECTIVES:**

Posterior fixation of the cervical spine (PFC) is commonly performed with rod-connected screws inserted in the vertebras. Because of the many sensitive structures located adjacent to the vertebras, computer-assisted navigation is often used with PFCs. We assessed safety and efficacy of free-hand placed PFCs.

**METHODS:**

We retrospectively identified all the patients in our institution between Januray 2006 and December 2016 who underwent a primary PFC for any indication. The postoperative images and medical records were evaluated for possible clinical failures, which was considered as a reoperation to cervical spine within 1 year after primary surgery for any reason, or a new screw-related adverse event including nerve root injury, spinal cord injury, or vertebral artery injury.

**RESULTS:**

In total, 332 patients and 1476 screws were included in the study. The evaluated screws were primarily pedicle and lateral mass screws. The rate of screw malposition was 12% (171/1476). The overall rate of clinical failure was 16% (52/332) and was significantly higher in patients with screw malposition than in those without malposition (24% vs 12%, *P* = .03). The rate of screw insertion–related clinical failure was 6.0% (20/332). The C7 pedicle seemed to be more prone to screw malposition than other locations (26/86, 30%).

**CONCLUSION:**

Clinical failure after posterior cervical spine fixation occurs significantly more frequently in patients with screw malposition compared with those without malposition. The overall rate of screw insertion–related clinical failure was 6.0%. These findings suggest that improving screw placement accuracy may reduce the risk of complications.

**ABBREVIATIONS:**
PFC: posterior fixation of the cervical spine
SRCF: screw insertion–related clinical failure.

Posterior fixation of the cervical spine (PFC) with a screw-rod technique is a common procedure performed in various conditions such as trauma, degenerative instability, or neoplasms. Various screw insertion techniques have been introduced in the literature.^1,2^ The most common sites for screw insertion within a target vertebra are the lateral masses and the pedicles. Pedicle screws are mainly used in the C2 and C7 vertebras and they have better pull-out strength compared with lateral mass screws.^3^ Depending on the level of the instability, the stabilization might extend to occipital bone or thoracic spine.

The anatomy of the cervical spine is complex with adjacent critical structures such as the spinal cord, the spinal nerve roots, and the vertebral arteries.^4^ The goal of the surgery was to provide a stable spine without complications such as vertebral artery injury, spinal cord injury, nerve root injury, or detachment of the fixation material over time. The fixation material has reached its purpose when the instrumented spinal segment has ossified properly. Therefore, a nonsymptomatic detachment of fixation material is sometimes considered an irrelevant finding.

The computer-assisted navigation is currently often used to improve safety and accuracy of screw placement.^2,5,6^ Earlier studies have shown that the screw malposition rate is significantly lower in navigated screws compared with free-hand inserted screws.^7,8^ However, the overall clinical benefit of 3-dimensional navigation in posterior fixation of the cervical spine remains unclear, as the rate of revision surgery due to screw malposition in the cervical spine is not well established.^5^ Moreover, cervical spine mobility during surgical manipulation and potential sources of error inherent to navigation systems mean that navigation does not guarantee accurate screw placement. Temporary technical malfunctions may also necessitate reliance on surgical expertise in free-hand screw placement. In addition, the availability of 3-dimensional navigation systems may be limited to a subset of operating rooms, and their high costs can further constrain widespread use. Therefore, navigation does not obviate mastery of free-hand screw placement.

In our hospital, most of the posterior cervical fixations have until recent years been performed without navigation. The aim of this study was to assess the safety and efficacy of posterior fixations in a large retrospective sample of consecutive patients with cervical spinal instability of any reason. We hypothesized that free-hand placement of screws is usually safe, and that screw malposition increases the rate of clinical failures, including reoperations within 1 year or significant screw-related adverse events. Our aim was to study in which instances the navigation should be used and when traditional anatomy based free-hand methods are sufficient.

## METHODS

All patients who underwent primary PFC at our institution for any indication between January 2006 and December 2016 and who had postoperative imaging available in the hospital records were retrospectively identified. All procedures were performed by experienced neurosurgeons. The Nordic Medico-Statistical Committee codes used for data search were NAJ12 = posterior reduction of fracture of cervical spine and NAG42 = posterior fusion of cervical spine with or without fixation.

### Radiological Data

**FIGURE 1.**
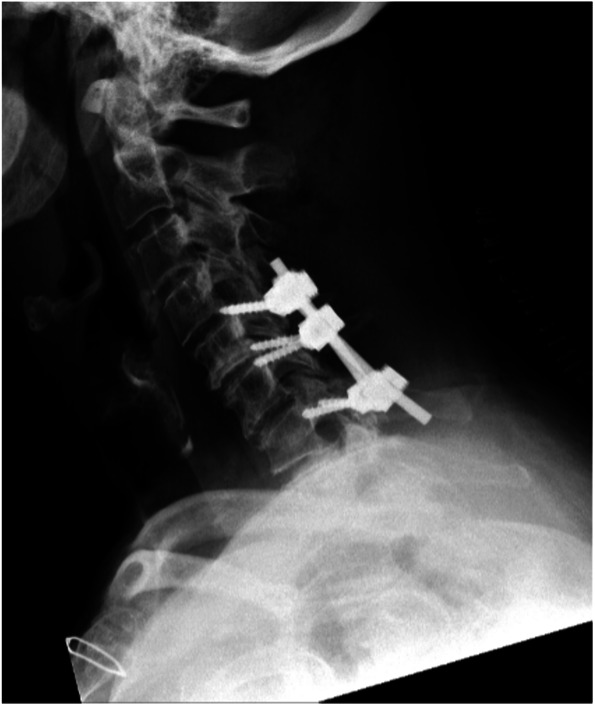
A postoperative x-ray of one of our patients shows posterior lateral mass fixation from C5 to C7. The precise evaluation of the screws' positions is difficult. However, screws seem to be inserted correctly.

**FIGURE 2.**
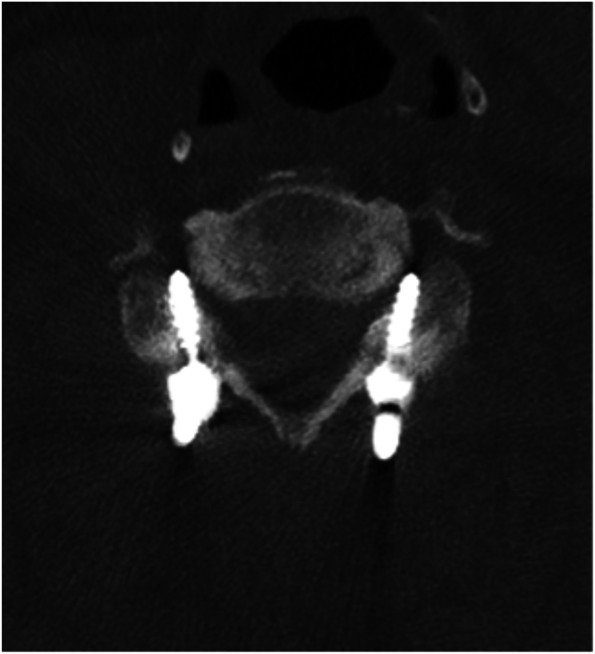
An axial computed tomography slice of the same patient at C6 level. Because of increasing nerve root symptoms, a computed tomography was taken. This slice shows lateral mass screws penetrating the nerve root channels bilaterally.

Imaging data consisted of postoperative x-ray (Figure 1) images and computer tomography (CT) images (Figure 2). Postoperative imaging consisted primarily of x-rays as part of routine follow-up. CT imaging was performed selectively based on clinical indication, such as suspected screw malposition or postoperative complications. Screw insertion sites were categorized as lateral mass, pedicle, and other (eg, intralaminar and translaminar screws).

The following categories were used for classifying screw placement accuracy: (1) accurate screw placement in the vertebra, (2) screw perforates the vertebra, (3) screw enters the nerve root canal, (4) screw enters the spinal canal, (5) screw enters the vertebral artery canal, and (6) undetermined screw placement. Class 1 includes intentionally placed bicortical screws (mainly lateral masses) with minimal cortical penetration that does not result in clearly measurable extension beyond the cortex and was not considered a malposition.

### Clinical Data

Data collected from the medical records included subject- and surgery-related data including indication for the surgery, reoperations, and time and length of the hospital admission.

Clinical failure was considered in 2 scenarios: (1) reoperation to cervical spine within 1 year after primary surgery for any reason and (2) new screw-related adverse event that did not need surgery. The following screw-related adverse events were used in the study: (1) nerve root injury, (2) spinal cord injury, and (3) vertebral artery injury.

Screw-insertion–related clinical failure (SRCF) was defined as a subset of clinical failures and was defined as a mechanical failure of the construct that led to revision surgery and could have been avoided by proper screw insertion, or a new screw-related adverse event.

SRCF may also occur during preparation of the screw canal before final screw placement. For example, transient contact with neural structures during drilling or tapping may result in neurological symptoms, even if the final screw position is subsequently correct. Similarly, a screw may initially be malpositioned during insertion and subsequently repositioned during the same procedure. Consequently, postoperative imaging may demonstrate well-positioned screws despite the occurrence of clinically relevant symptoms related to the insertion process.

Occipital screw and lamina hook detachments were excluded, as well as rod-related detachments and rod-related poor postsurgery postures as they could not have been avoided with more precise placement of the cervical screws. If the surgery was performed properly as planned, and the fixation was intact, but the postsurgery result was considered insufficient, the primary surgery was considered successful considering screw placements.

The medical records and imaging studies (x-ray and CT) of all patients were reviewed independently by 2 medical students, V.K. and E.Y., especially trained to assess screw positioning by the last author TTh, who also reviewed all those cases in which the independent primary assessment did not reach consensus. The mean follow-up time was 7.1 years (IQR 4.7 years, min 2.6 years, max 13.4 years).

This study was performed based on retrospective evaluation of clinical records and imaging studies. Therefore, an approval by the ethics committee was not needed. The study was conducted according to the principles of Declaration of Helsinki.

As the study was purely retrospective and the patients were not contacted directly, informed consent was not required.

### Statistics

Nominal and ordinal variables are presented as ratios and percentages, and the Fisher exact test or χ^2^ test used as appropriate to compare them statistically. Odds ratios (OR) with 95% CI were calculated for clinically relevant associations. For continuous variables, median and IQR is given and the Mann-Whitney *U*-test was used for statistical comparison. *P*-values <.05 were considered as significant. Statistics were calculated using IBM SPSS software (IBM Corp.).

## RESULTS

**FIGURE 3.**
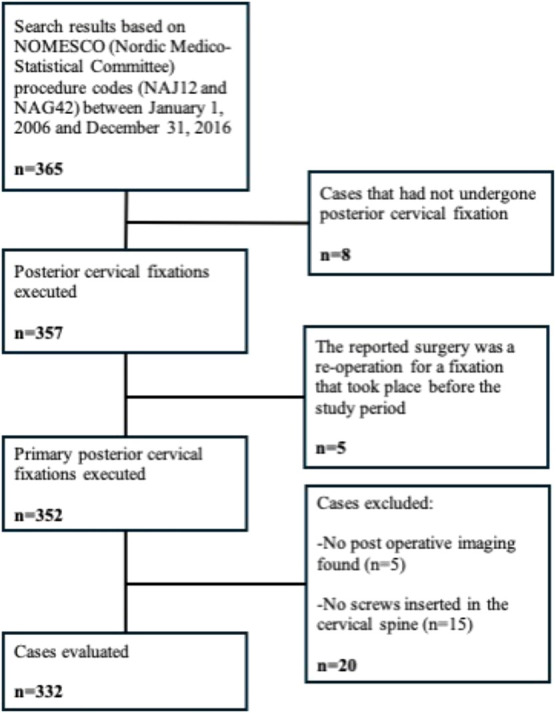
Flowchart presenting the patient selection in the study.

A total of 332 patients were included in the study. Flowchart (Figure 3) presents the inclusion/exclusion of the patients. The demographic characteristics of the patients is presented in Table 1 and CT-cohort in Table 2. For comparison, the characteristics of the patients with SRCF (n = 20) are presented in parallel with patients without SRCF (n = 312) and the whole sample (n = 332).

**TABLE 1.** Demographics and Clinical Characteristics of the Patients Treated With Posterior Fixation of the Cervical Spine From January 2006 to December 2016 Stratified According to the Possible SRCF

Clinical variable	SRCF^a^		*P*-value	Total 332
	Yes 20/332	No 312/332		
Age at surgery, median (IQR)	68 (58-75)	67 (57-75)	*.883*	67 (57-75)
Gender (% of women)	30% (6/20)	29% (92/312)	.961	30% (98/332)
Indication for surgery				
Fracture	65% (13/20)	71% (222/312)	.716	71% (235/332)
Degenerative	25% (5/20)	22% (70/312)		23% (75/332)
Tumor	5.0% (1/20)	4.8% (15/312)		4.8% (16/332)
Other	5.0% (1/20)	1.6% (5/312)		1.8% (6/332)
Time of primary hospitalization, median days (IQR)	6 (3-8)	5 (3-7)	.818	5.0 (3-7)^b^
Radiological outcome				
1. Accurate screw placement in all screws in the vertebra	35% (7/20)	65% (204/312)	.01	64% (211/332)
2. At least one screw position undesired	65% (13/20)	32% (100/312)		34% (113/332)
3. At least one screw placement undetermined without undesired positions	0%	2.6% (8/312)		2.4% (8/332)
Navigation-assisted operation	5% (1/20)	3.8% (12/312)		3.9% (13/332)
SRCF^a^				6.0% (20/332)
Reoperation due to screw inside the spinal canal	15% (3/20)			0.9% (3/332)
Nerve root injury	20% (4/20)			1.2% (4/332)
Reoperation due to detachment of the fixation	45% (9/20)			2.7% (9/332)
Screw inserted at wrong vertebra	10% (2/20)			0.6% (2/332)
Reoperation due to inadequate and painful fixation	10% (2/20)			0.6% (2/332)

SRCF, screw-insertion–related clinical failure.

a SRCF was defined as a subset of clinical failures and was defined as a mechanical failure of the construct that led to revision surgery and could have been avoided by proper screw insertion or a new screw-related adverse event.

b One patient's length of hospitalization was missing.

**TABLE 2.** Demographics and Clinical Characteristics of the Patients Treated With Posterior Fixation of the Cervical Spine From January 2006 to December 2016 and Imaged Postoperatively With Computer Tomography Stratified According to the Possible SRCF

Clinical variable	SRCF^a^		*P*-value	Total 113
	Yes 12/113	No 101/113		
Age at surgery, median (IQR)	69 (61-77)	68 (57-76)	.538	68 (57-76)
Gender (% of females)	25% (3/12)	20% (20/101)	.672	20% (23/113)
Indication for surgery				
Fracture	75% (9/12)	81% (82/101)	.624	81% (91/113)
Degenerative	25% (3/12)	16% (16/101)		17% (19/113)
Tumor	0%	3.0% (3/101)		2.7% (3/113)
Other	0%	0%		0%
Time of primary hospitalization, median days (IQR). [range]	6 (4-8)	6 (4-8)	.937	6.0 (4-8)^b^
Radiological outcome				
1. Accurate screw placement in all screws in the vertebra	17% (2/12)	39% (39/101)	*.140*	36% (41/113)
2. At least one screw position undesired	83% (10/12)	60% (61/101)		63% (71/113)
3. At least one screw placement undetermined without undesired positions	0%	1% (1/101)		0.9% (1/113)
Navigation-assisted operation	0% (0/12)	8.9% (9/101)	.281	8.0% (9/113)
SRCF^a^				11% (12/113)
Reoperation due to screw inside the spinal canal	17% (2/12)			1.8% (2/113)
Screw inside nerve root canal and/or nerve root injury	33% (5/12)			4.4% (5/113)
Reoperation due to detachment of the fixation	33% (4/12)			3.5% (4/113)
Reoperation due to inadequate and painful fixation	8.3% (1/12)			0.9% (1/113)

SRCF, screw-insertion–related clinical failure.

a SRCF was defined as a subset of clinical failures and was defined as a mechanical failure of the construct that led to revision surgery and could have been avoided by proper screw insertion, or a new screw-related adverse event.

b One patient's length of hospitalization was missing.

### Radiological Results of Screw Placements

Postoperative imaging was available in all included cases: X-ray (Figure 1) in 219 cases (66%) and CT (Figure 2) in 113 cases (34%). A total of 1476 screws were inserted into the cervical spines of the studied 332 patients and evaluated in this study.

Most of the screws were inserted either in the lateral mass (n = 1058, 72%) or the pedicle (n = 326, 22%) of the target vertebra. Some translaminar or transarticular screws (6.2%, n = 92) were also used, mainly at C2 level (n = 79, 86%). Additional laminar hooks were used in 54 patients (16%). Fixation was extended in the thoracic spine in 61 patients (18%) and to the skull in 61 patients (18%).

The number of screws in different positions based on the whole sample was as follows: accurate screw placement in the vertebra, n = 1283 (87%); screw perforates the vertebra, n = 122 (8.3%); screw enters the nerve root canal, n = 29 (2.0%); screw enters the spinal canal, n = 14 (0.9%); screw enters the vertebral artery canal, n = 6 (0.4%); and undetermined screw placement, n = 22 (1.5%).

The accuracy of the screw placement is presented in Table 3. The risk for malposition of the screw was higher in the pedicles, n = 50 of 326 (15%) compared with the lateral masses, n = 108 of 1058 (11%). The lowest risk of undesired screw insertion occurred at the first cervical level (atlas), affecting 3 of 137 screws (2.1%), with particularly low rates in the lateral mass (1/121, 0.8%). The highest risk was observed at C7, with 39 of 166 screws (23%), most frequently in the pedicle (26/86, 30%).

**TABLE 3.** Screw-Insertion Sites and Accuracy

Clinical variable	Desired screw position			Total 1476
	No (n = 171)	Yes (n = 1283)	Undetermined (n = 22)	
Site of screw insertion				
Lateral mass	10.2% (108/1058)	88.1% (932/1058)	1.7% (18/1058)	71.7% (1058/1476)
Pedicle	15.3% (50/326)	84.0% (274/326)	0.6% (2/326)	22.1% (326/1476)
Other	14.1% (13/92)	83.7% (77/92)	2.1% (2/92)	6.2% (92/1476)
Total	11.6% (171/1476)	86.9% (1283/1476)	1.5% (22/1476)	1476
Level of screw insertion				
Atlas (C1)	2.1% (3/137)	97.1% (133/137)	0.7% (1/137)	9.3% (137/1476)
Lateral mass	0.8% (1/121)	98.3% (119/121)	0.8% (1/121)	88.3% (121/137)
Pedicle (through posterior arch)	10% (1/10)	90% (9/10)	0	7.3% (10/137)
Other	16.7% (1/6)	83.3% (5/6)	0	4.4% (6/137)
Axis (C2)	11.1% (32/306)	88.2% (270/306)	0.6% (2/306)	20.7% (306/1476)
Lateral mass	22.2% (2/9)	77.8% (7/9)	0	2.6% (9/306)
Pedicle	9.6% (21/218)	89.4% (195/218)	0.9% (2/218)	71.2% (218/306)
Other	13.9% (11/79)	86.1% (68/79)	0	25.8% (79/306)
C3	9.9% (24/243)	89.3% (217/243)	0.8% (2/243)	16.5% (243/1476)
Lateral mass	9.7% (23/238)	89.5% (213/238)	0.8% (2/238)	98.0% (238/243)
Pedicle	0	100% (3/3)	0	1.2% (3/243)
Other	50% (1/2)	50% (1/2)	0	0.8% (2/243)
C4	10.1% (22/217)	88.0% (191/217)	1.8% (4/217)	14.7% (217/1476)
Lateral mass	9.9% (21/213)	88.3% (188/213)	1.9% (4/213)	98.2% (213/217)
Pedicle	25% (1/4)	75% (3/4)	0	1.8% (4/217)
Other	0	0	0	0
C5	9.7% (20/206)	87.4% (180/206)	2.9% (6/206)	14.0% (206/1476)
Lateral mass	9.9% (20/203)	88.2% (179/203)	2.0% (4/203)	98.5% (203/206)
Pedicle	0	100% (1/1)	0	0.5% (1/203)
Other	0	0	100% (2/2)	1.0% (2/203)
C6	14.4% (29/201)	83.1% (167/201)	2.5% (5/201)	13.6% (201/1476)
Lateral mass	14.2% (28/197)	83.2% (164/197)	2.5% (5/197)	98.0% (197/201)
Pedicle	25% (1/4)	75% (3/4)	0	2.0% (4/201)
Other	0	0	0	0% (0/201)
C7	23.5% (39/166)	75.3% (125/166)	1.2% (2/166)	11.2% (166/1476)
Lateral mass	16.9% (13/77)	80.5% (62/77)	2.6% (2/77)	46.4% (77/166)
Pedicle	30.2% (26/86)	69.8% (60/86)	0	51.8% (86/166)
Other	0	100% (3/3)	0	1.8% (3/166)

In the CT-based data, malposition rates were generally higher, particularly for pedicle screws at C2 and C7 (Table 4). The malposition rate for C1 screws remained low in both data sets.

**TABLE 4.** Screw-Insertion Sites and Accuracy in Computer Tomography–Imaged Cases

Clinical variable	Desired screw position			Total 542
	No 21.8% (118/542)	Yes 77.9% (422/542)	Undetermined 0.4% (2/542)	
Site of screw insertion				
Lateral mass	17.2% (73/424)	82.8% (351/424)	0% (0/424)	78.2% (424/542)
Pedicle	35.5% (38/107)	64.5% (69/107)	0% (0/107)	19.7% (107/542)
Other	63.6% (7/11)	18.2% (2/11)	18.2% (2/11)	2.0% (11/542)
Level of screw insertion				
Atlas (C1)	4.5% (1/22)	95.5% (21/22)	0	4.1% (22/542)
Lateral mass	5.6% (1/18)	94.4% (17/18)	0	81.8% (18/22)
Pedicle (through posterior arch)	0	100% (4/4)	0	18.2% (4/22)
Other	0	0	0	0
Axis (C2)	47.5% (19/40)	52.5% (21/40)	0% (0/40)	7.4% (40/542)
Lateral mass	0	0	0	0
Pedicle	37.5% (12/32)	62.5% (20/32)	0	80.0% (32/40)
Other	87.5 (7/8)	12.5% (1/8)	0	20.0% (8/40)
C3	24.5% (13/53)	75.5% (40/53)	0% (0/53)	9.8% (53/542)
Lateral mass	25% (13/52)	75% (39/52)	0	98.1% (52/53)
Pedicle	0	0	0	0
Other	0	100% (1/1)	0	1.9% (1/53)
C4	13.7% (10/73)	86.3% (63/73)	0	13.5% (73/542)
Lateral mass	13.7% (10/73)	86.3% (63/73)	0	100 (73/73)
Pedicle	0	0	0	0% (0/73)
Other	0	0	0	0% (0/73)
C5	13.0% (15/115)	85.2% (98/115)	1.7% (2/115)	21.2% (115/542)
Lateral mass	13.3% (15/113)	86.7% (98/113)	0	98.2% (113/115)
Pedicle	0	0	0	0
Other	0	0	100% (2/2)	1.7% (2/115)
C6	19.3% (23/119)	80.1% (96/119)	0% (0/119)	22.0% (119/542)
Lateral mass	19.0% (22/116)	81.0% (94/116)	0	97.5% (116/119)
Pedicle	33.3% (1/3)	66.7% (2/3)	0	2.5% (3/119)
Other	0	0	0	0
C7	30.8% (37/120)	69.1% (83/120)	0% (0/120)	22.1% (120/542)
Lateral mass	23.0% (12/52)	77.0% (40/52)	0	43.3% (52/120)
Pedicle	36.8% (25/68)	63.2% (43/68)	0	56.7% (68/120)
Other	0	0	0	0

### Clinical Results of the Posterior Fixations

Altogether 52 of 332 patients (16%) experienced a clinical failure after the primary operation. The reasons for clinical failures were as follows: infections, n = 9 (2.7%); screw detachment from the cervical spine, n = 9 (2.7%); stable but inadequate fixation that led to a reoperation, n = 5 (1.5%); anterior cervical spine fixation within 1 year post primary surgery, n = 4 (1.2%), post-operative hematoma, n = 4 (1.2%); occipital screw detachment, n = 4 (1.2%); nerve root injury, n = 4 (1.2%); screw inside the spinal canal, n = 3 (0.9%); screw inserted into wrong vertebra, n = 2 (0.6%); reoperation to remove the fixation material after ossification due to nonspecific pain and motion restriction, n = 2 (0.6%); reoperation caused by inadequate and painful fixation, n = 2 (0.6%); reoperation for taking a biopsy from a tumor found during the primary operation, n = 1 (0.3%); undesired cervical rods alignment, n = 1 (0.3%); rod detached from the screw, n = 1 (0.3%); and hooks detached from the vertebra, n = 1 (0.3%). There were no vertebral artery injuries nor spinal cord injuries.

### Association Between Clinical Failures and Screw Malposition

In the whole data set, clinical failure was detected in 27 of 113 patients (24%) with one or more misplaced screws, compared with 25 of 211 patients (12%) with no misplaced screws (*P* = .03; OR 2.22, 95% CI 1.22-4.04). In the CT-based data, clinical failure was detected in 21 of 71 patients (30%) with one or more misplaced screws, compared with 10 of 41 patients (24%) with no misplaced screws (*P* = .554).

### Association Between SRCF and Screw Malposition

Of the 52 clinical failures, 20 (39%) were considered as SRCF representing a mechanical failure of the construct that led to revision surgery or a new screw-related adverse event (Table 1).

The rate of SRCF was 6.0% (20/332). Overall, in the whole data set, SRCF was detected in 13 of 113 patients (12%) with one or more misplaced screws, compared with 7 of 211 patients (3.3%) with no misplaced screws (*P* = .003, OR 3.94, 95% CI 1.52-10.17). In the CT-based data, SRCF was detected in 10 of 71 patients (14%) with one or more misplaced screws, compared with 2 of 41 patients (4.9%) with no misplaced screws (*P* = .140).

The length of stay in hospital after primary surgery did not significantly differ between those who experienced SRCF and those who did not (6 vs 5 days, *P* = .818).

## DISCUSSION

In this study, we describe the accuracy of free-hand screw placement in the cervical spine in a consecutive single center patient series. The overall risk of clinical failure was 52 of 332 (16%) and the risk of SRCF was 20 of 332 (6.0%).

### Accuracy of Screw Insertion in the Cervical Spine

Previous literature reports screw malposition rates in the cervical spine between 0% and 50%, although there is some variety in the malposition grading methods, which makes it difficult to compare screw insertion accuracy between studies.^2,3,5-10^ There is abundance of literature showing that navigation increases the rate of screw insertion accuracy in the spine.^2,5-8,10^ It is widely accepted, however, that inserting screws in the lateral masses of the cervical spine is safe without navigation assistance. The pedicles in the cervical spine are small with adjacent critical structures which warrants the use of navigation assistance. For example, in the systematic review by Mahmoud et al,^8^ the cervical pedicle screw accuracy rate by free-hand method was 81% and by navigation assistance 91%. However, there are also studies that have contradictory results.^9^

In our study, malposition rate of 15% (36% in CT-based data) (Table 4) for pedicle screws is high. One reason for this might be our strict assessment of screw positions. Postoperative CT was mainly performed when there was a clinical suspicion of screw malposition or a clinical failure. Therefore, in the CT-based sample, the rate of malpositions is higher than that would be expected if the whole sample was CT imaged. On the other hand, the high number of C7 pedicle screw malpositions compared with more cranial spinal segments might also be explained by higher usage of postoperative CT imaging with C7 screws. In our study, screws were evaluated from CT images in 72% (65/90) of the cases that had C7 screws inserted, whereas in the whole sample, only 34% (113/332) of the cases were evaluated from a CT image.

### Screw Misplacement and Clinical Failures

In total, we found clinically relevant failures in 16% (52/332) of the cases. Most of the clinical failures were not related to screw insertion accuracy. In addition, radiological screw malpositions occurred in 12% of all screws, whereas SRCF occurred in only 6.0% of patients. This suggests that a substantial proportion of radiological malpositions are clinically irrelevant. However, malpositioned screws were associated with a 3-fold increase in the risk of clinical failure (14% vs 4.9% on CT-based data, 12% vs 3.3% on the whole dataset). The sex, age, or indication of the primary surgery did not affect the rate of SRCFs (Table 1).

### Navigated Screws

Navigation did not reduce the number of clinically significant screw misplacements. However, total number of the navigated cases was too low (13/332 (3.9%)) for drawing a conclusion. A meta-analysis by Verma et al^6^ published in 2010 includes and compares 23 studies with a total of 5992 spinal pedicle screws. They did not find any functionally relevant differences in outcomes between navigation-assisted and conventional techniques, when looking at pedicle screws inserted in any spinal level. On the other hand, a systematic review by Lange et al^5^ compares 24 studies with a total of 5207 cervical pedicle screws. They suggest that by increasing the accuracy of screw placement, navigation might increase the safety of pedicle screw insertion in the cervical spine and would therefore be a recommended policy. They base this conclusion also on the fact that navigation reduces radiation for the patient and surgical staff by removing the need for intraoperative x-ray imaging. In a subgroup analysis of their systematic review and meta-analysis published in 2023, Soliman et al^11^ found that significantly fewer complications occur with navigated patients. Yet, when looking at individual complications, no significant difference was found. Their material consisted of 4165 patients and 16 669 pedicle and lateral mass screws.

### Implications for Clinical Practice

In our data set, one-third of the C2 and C7 pedicle screws were misplaced despite the operator being experienced. Therefore, we conclude that navigation would add value in cervical pedicle screw placement. Given that one-fifth to one-seventh of the lateral mass screws (whole sample vs CT imaged) were also misplaced, navigation merits to be considered also in lateral mass screw placement.

In our data set, higher rates of screw malposition were observed in the CT-imaged cohort, likely reflecting the better accuracy of CT for detecting malpositions. Given the increased risk of clinical failure when one or more screws are misplaced, false negative for screw misplacement should be avoided. Therefore, based on our data, CT imaging should be considered for postoperative control after posterior fixation of the cervical spine.

### Strengths and Limitations of the Study

This study is based on a large sample of unselected PFC patients, with long-term follow-up. Procedures are centralized within a limited number of tertiary referral centers, and patients are typically treated within defined catchment areas. Consequently, most follow-up procedures and postoperative complications are managed at the same institution where the primary operation was performed, ensuring comprehensive follow-up.

Only one-third of the cases were evaluated from postoperative CT, which is a significantly more precise imaging modality than x-ray for postoperative assessment. Therefore, the screw malposition rate and the association between the imaging finding and clinical failures could be different if a postoperative CT was available for all cases. In addition, the selective use of CT imaging may have introduced selection bias, as CT was often performed in cases with clinical suspicion of screw malposition.

Computer-assisted navigation was used in only 13 of 332 cases, which precluded a meaningful comparison between free-hand and navigation-assisted screw placement. Consequently, only indirect conclusions regarding the clinical benefit of navigation can be drawn from the present data.

Sometimes defining whether a screw inserted in the C2 is a pedicle screw or a pars screw is not that straightforward. Some of the C2 pars screws were possibly classified as pedicle screws especially from x-ray images. In addition, most likely, the 9 screws at C2 level classified as lateral mass screws, could have been considered as pars or pedicle screws. Different classification in these cases would not have, however, changed our conclusions.

Some of the reoperations within 1 year might have been de novo pathologies and not necessarily related to the primary surgery. Therefore, the number of actual clinical failures might be lower than reported. In addition, we did not include minor complications such as superficial wound infections in our analysis.

## CONCLUSIONS

Clinical failure after posterior cervical spine fixation occurred significantly more frequently in patients with screw malposition compared with those without malposition. The overall rate of screw insertion–related clinical failure was 6.0%. These findings suggest that improving screw placement accuracy may reduce the risk of complications.

## COMMENTS

In this article, the authors present a retrospective review of patients who underwent posterior cervical fixation with screws and rods, assessing the safety of free-hand screw placement using postoperative radiographs and computed tomography (CT) scans. The study highlights an important distinction between radiographic screw malposition and clinically relevant screw-insertion-related failure. Although radiographs are less sensitive than CT scans for detecting screw malposition and may overestimate placement accuracy, the radiograph-only cohort still provides clinically meaningful outcome data.

Notably, screw-insertion-related clinical failure occurred in only a small minority of patients evaluated with radiographs alone, including those with screw placement deemed accurate. Similarly, among patients with screw positions assessed as undesirable on radiographs and CT scans combined, only a small proportion developed such failure. Although accurate assessment of screw placement remains important, these findings reinforce the widely held view that not all radiographic malpositions are clinically consequential. Overall, the study provides useful clinical evidence supporting the safety of free-hand posterior cervical screw fixation when performed with appropriate technique.

**Femi Owagbemi and Wale Sulaiman**

*Lagos, Nigeria*
